# Retraction: Effect of different anesthetic methods on cellular immune functioning and the prognosis of patients with ovarian cancer undergoing oophorectomy

**DOI:** 10.1042/BSR-2017-0915_RET

**Published:** 2022-08-03

**Keywords:** cellular immune function and prognosis, Combined general/epidural anesthesia, general anesthesia, ovarian cance

This article is being retracted from *Bioscience Reports* at the request of several listed co-authors who claim to have had no involvement in the paper and that they were unaware of its submission and publication. The corresponding author has been contacted with regards to the retraction and has not responded to the Journal's queries or the concerns raised. Several listed co-authors wish to retract the article and the Editorial Board agree with the Retraction.

